# Community-Based Mental Health Care in Britain

**DOI:** 10.17650/2712-7672-2020-1-2-14-20

**Published:** 2020-12-04

**Authors:** Tom Burns

**Affiliations:** Department of Psychiatry, University of Oxford, Warneford Hospital

**Keywords:** Community Mental Health Teams, sectorization, functional teams, general practice, амбулаторные психиатрические бригады, секторальный принцип, медицинская помощь, функциональные бригады, общая врачебная практика

## Abstract

Community mental health care in the UK was established by two influential mental health acts (MHAs). The 1930 MHA legislated for voluntary admissions and outpatient clinics. The 1959 MHA required hospitals to provide local follow- up after discharge, required them to work closely with local social services and obliged social services to help with accommodation and support. An effect of this was to establish highly sectorized services for populations of about 50,000. These were served by multidisciplinary teams (generic CMHTs), which accepted all local referrals from family doctors. Sector CMHTs evolved a pragmatic approach with an emphasis on skill-sharing and outreach, depending heavily on community psychiatric nurses. The NHS is funded by central taxation, with no distortion of clinical practice by per-item service fees. It is highly centrally regulated, with a strong emphasis on evidence-based treatments.

Since 2000, generic sector teams have gradually been replaced or enhanced by Crisis Resolution Home Treatment teams, Assertive Outreach Teams and Early Intervention Teams. Assertive Outreach Teams were resorbed into CMHTs, based on outcome evidence. The last decade has seen a major expansion in outpatient psychotherapy (Improving Access to Psychological Treatments (IAPT) services) and in specialist teams for personality disorders and perinatal psychiatry. The traditional continuity of care across the inpatient-outpatient divide has recently been broken. During the last decade of austerity, day care services have been decimated, and (along with the reduction in availability of beds) compulsory admission rates have risen sharply. Mental health care is still disadvantaged, receiving 11% of the NHS spend while accounting for 23% of the burden of disease.

## WHEN WAS COMMUNITY-BASED CARE ESTABLISHED?

The United Kingdom of Great Britain and Northern Ireland (hereafter referred to as the UK) was in the vanguard of the asylum movement in the early 19th century. Following the example set by the Quaker Tuke family in the York Retreat in 1796, asylums based on 'moral therapy' were established throughout the nation from the early 1800s. These remained the dominant model of psychiatric care for psychotic illnesses until 1930. Strict legislation in the late 19th century, to protect the rights of detained patients, had an unintended consequence of hindering early intervention and flexible care.

The predominantly degenerative view of mental illness was finally shaken by experiences in the First World War and the succeeding decades. Shell-shocked soldiers confirmed the involvement of psychological processes in the causation and treatmeet of mental illnesses. The dramatic success of malaria treatment for general paralysis of the insane (GPI) and promising early sleep therapies brought psychiatry closer to medicine and began to erode the isolation of the asylums. There had been many individual initiatives prior to this. The first psychiatric outpatient clinics were established at St Thomas's in London and in the Wakefield asylum in 1890, along with hostels for discharged patients (such as that in Dingleton Hospital in 1880) and general hospital outpatient clinics in Portsmouth in 1926. However, these early experiments failed to catch on. The modern era was ushered in with the establishment of the Maudsley Hospital in 1923, devoted to short-term care, and the 1930 Mental Treatment Act.

## THE 1930 MENTAL TREATMENT ACT AND THE 1959 MENTAL HEALTH ACT

Community mental health in the UK was effectively established by these two acts. The 1930 act permitted voluntary admissions, outpatient care in mental hospitals and for local authorities (who, at that time, were responsible for all mental health care) to spend money on supporting discharged patients. It also changed terminology from 'asylum' and 'lunatic' to 'mental hospital' and 'mental patient'. This more outward-looking attitude witnessed slow, but informal, growth in outpatient care. The first day hospitals and day centres were opened after WWII, beginning with the Marlborough Day Hospital in London, opened by Joshua Beirer in 1946. In 1948, the National Health Service was established, and mental health care was transferred to it from local authorities. This amalgamation cast into stark relief the contrast between the flexibility of general healthcare and that for mental health, and it prompted much soul-searching.

The development of UK community mental health care as it is now known can be traced to the 1959 MHA. The 1959 MHA reflected the optimistic spirit of its age and the impact of the post-war welfare state, which guaranteed basic financial security for disabled citizens. As well as introducing strict regulations for the use and monitoring of compulsory care, the 1959 MHA contained two specific provisions which profoundly shaped developments. Firstly, the act placed local authority social services at the centre of care for the severely mentally ill. Social workers had authority over compulsory admissions (albeit on the recommendation of psychiatrists). The act also legislated both the resources and the obligation to provide aftercare. Secondly, the act required any hospital that took in detained patients to, itself, provide them with outpatient follow-up and aftercare. To achieve these ends (both cooperation over compulsory admissions and outpatient follow-up), mental hospitals had to develop close working relationships with local authority social workers.

The only practical way to achieve such close working relationships was by establishing catchment areas and, eventually, sectorization. Unlike the French, whose 'secteur' was centrally dictated, UK sectorization (manifest in the growth of local Community Mental Health Services - CMHTs) grew organically as a pragmatic response to these requirements. CMHTs spread in reach and sophistication throughout the 1960s and 1970s and became the default structure for community care until the radical changes introduced in 1999.

## THE GENERIC CMHT

By the late 1970s, most of the UK population accessed its specialist mental health care via a generic CMHT [1]. Teams served a defined population and were expected to assess anyone referred to them irrespective of diagnosis or severity of disorder. The populations served initially numbered about 50,000, but this number has shrunk as resources and specialization have increased. The team was responsible for all outpatient and inpatient care, usually having access to a number of beds in the local psychiatric unit. Psychiatry in the UK is explicitly a secondary service. Virtually all patients, other than the homeless and those in chaotic inner-city situations, are referred after assessment by their family doctor.

The traditional CMHT is multidisciplinary, comprising, at a minimum, psychiatrists, community psychiatric nurses and social workers. It may also include occupational therapists, psychologists, health care assistants and sometimes other specialists. It is headed by a specialist psychiatrist, and Community Psychiatric Nurses (CPNs) are usually its most numerous members (2-5). CPNs were developed in 1953 as a fledgling service to monitor discharged psychosis patients [2] but have long outnumbered all other community MH staff [3]. They are the case managers for most patients, usually carrying a caseload of 20-30 patients, with contact monthly or more often, as needed. CMHTs accept all referrals from family doctors so must deal with the whole range of disorders, from long-term psychoses to short-term crises, anxiety and depression. Managing referrals to match expectations and capacity has always been a challenge for CMHTs, and this has become increasingly so, often with an explicit focus on patients with a severe mental illness (SMI). CMHTs are usually based in some form of shared community centre, and outreach has been a central feature of practice. In the case of CPNs in particular, most of their contact with patients is home- based, and this practice is common in other disciplines.

The population served by each CMHT was initially geographically defined but has increasingly been based on general practice lists. This strengthens working relationships and continuity between primary and secondary care. Such comprehensive responsibility for a clearly defined population powerfully focuses CMHTs on the most seriously ill patients. Because difficult patients cannot be declined or sent elsewhere, UK mental health care is pragmatic, with little scope for rigid theories. The care provided is, of necessity, eclectic, and there is a long tradition of role blurring and skill sharing.

CPNs are the backbone of the service and the group primarily responsible for monitoring and supporting psychosis patients, often administering long-acting antipsychotic medications. Social workers have specific responsibilities for ensuring accommodation and financial support. Psychologists, where they are present, often take the lead in psychotherapy and talking treatments.

UK CMHTs have strikingly informal working practices. Titles are rarely used; first name terms are the rule, and professional boundaries are not defended. All members spend much of their time on supportive social care. For example, a nurse or psychologist would not hesitate to ring up a housing department. Initial assessments are not always conducted by medical staff where nurses and psychologists have taken prominent roles. This nonhierarchical style was inherited from the therapeutic community movement that was so influential when CMHTs were beginning [4].

The extent of role-blurring may also be a consequence of the NHS funding system, with the absence of any 'fee for service' or targeted payments. NHS MH services are funded by a relatively simple block grant. This is based on a capitation formula, plus sophisticated adjustments for levels of deprivations. In recent years, commissioning of services has become more localized, with specific targets for individual services.

## SPECIALIST AND FUNCTIONAL TEAMS

CMHTs have always been stratified by age group. Alongside the adult service (for 18-65-year-olds), parallel services were established very early for children and adolescents (up to the age of 18) and old-age services for those over 65. The structure and functioning are essentially similar for all three sets of teams, although the populations served vary. In addition, most regions had specialized teams that the CMHTs could refer to. There were liaison teams in hospitals, forensic services for mentally disordered offenders, and rehabilitation teams for severely and chronically disabled patients. Depending on local resources, there might also be specialized teams for eating disorders and personality disorders, although these were not universal.

These UK community MH services evolved organically through the 1960s to the 1990s. In the 1990s, however, evidence-based practice (ushered in with Stein and Test's study of ACT) [5] began to impact on planning, which became more centralized and specific. In 1999, the National Service Framework for Mental Health [6] proposed replacement of generic CMHTs by four specific services ('functional teams'). These were a Home Treatment Crisis Resolution (HTCR) team to deflect admissions, an Assertive Outreach Team (AOT - essentially an ACT team) to support 'revolving door' psychosis patients, an Early Intervention Service (EIS) for first-episode psychosis and, lastly, a Primary Care Liaison Team (PCLT) for everything else. PCLTs did not survive, and the other functional teams have gradually been rolled out nationally alongside generic CMHTs.

AOT teams were the first functional teams to be introduced. They were subjected to rigorous research and found not to be an improvement on CMHTs [7,8], so their functions have been resorbed back into CMHTs. The other two specialist teams have not been subjected to anything like the same rigorous research and remain central features of current practice. The provision of standalone personality disorder services [9] is now nationwide, and liaison services in general hospitals have been significantly enhanced. A striking (and unevidenced) recent development has been the splitting of community teams from inpatient responsibilities (the so-called 'functional split'). This arose from concerns about the quality of inpatient care. However, loss of continuity and unanticipated complexities have led to doubts over its wisdom [10].

## PSYCHOTHERAPY SERVICES (IAPT)

UK mental health has had very little private care provision. Apart from some very limited access to psychoanalysis, there has been no tradition of private psychotherapy as in other European countries. Simple psychotherapy has long been available within the NHS, and in the 1970s, psychotherapy was already recognized as a subspecialization within psychiatry, with its own training requirements. While this was intended to protect psychotherapy and its training, it also served to isolate it somewhat. Clinical psychologists in the NHS have increasingly expanded their expertise in, and responsibility for, cognitive behaviour therapy. This is now the primary evidence-based psychotherapy recommended by NICE (the National Institute for Clinical Excellence). In 2007, a separately funded provision (based on a stepped-care model) was introduced, called Increased Access to Psychological Treatments (IAPT) [11]. This programme provided advice and self-care but also trained CBT therapists to provide more intensive treatment, initially from primary care. Once established, the service was absorbed into secondary MH care and is now routine. It has ensured much greater access to psychotherapy, with an estimated additional 3000 staff nationally. However, it has been criticized by some for its restriction to CBT, its rather rigid format and the quality of therapist training.

## FINANCING AND LEGAL STRUCTURE

UK mental health care is funded by general taxation via the NHS and is totally free at the point of service. There are no patient-level payments (no itemized payments), although some service-level targets may affect funding.

Currently, it accounts for 11% of the total NHS spend (9.8% of GDP, in line with the EU average of 9.7% in 2014). In 2012, the Health and Social Care Act committed to 'parity of esteem' between physical and mental health care by 2020. As mental disorders account for 23% of the burden of disease, there is clearly quite some way to go. Local structures for setting priorities can have significant effects at the margins, but NHS MH services are generally fairly consistent nationally and remain subject to no complex financial distortions of clinical practice.

The 1959 MHA was revised in 1983 and again in 2007, and it is currently undergoing another revision. The UK is out of step with most of Europe in that compulsory admissions and treatment are initiated clinically rather than by a legal decision. They are subject to routine legal ratification of their justification by tribunals at set intervals, but this arrangement is subject to strong criticism and may change. Compulsory admissions are for set maximum periods (one month or six months and, in rare emergencies, three days), with legal representation available for patients at tribunals. The 2007 revision made two major changes. Firstly, it removed the four categories of disorder (mental illness, learning disability, severe learning disability and personality disorder) and replaced them with a single category of mental disorder. The rationale for this was to remove a 'treatability' clause that had been introduced into the 1959 MHA for personality disorder. Now, all patients are detained on the grounds of risk to health and the availability of 'appropriate treatment' (which is very broadly interpreted and can include care and supervision). The second major change was to introduce community treatment orders (CTOs). These are targeted on revolving door psychosis patients to ensure continued follow-up and maintenance medication. They are for six months in the first instance, after discharge from an involuntary admission, and are renewable for one year at a time without limit. Despite the evidence that they provide no benefit for patients [12,13], about 4000 per year are imposed.

The rate of compulsory admissions in the UK is 114 per 100,000. This is in the mid-range for Europe, with Austria highest at 282 and Italy lowest at 14.5 [14]. However, there are two significant issues of concern. The first is that the rate is rising faster than that of most comparable countries, and the second is the persistently high rate of detention among black patients. The number of compulsory admissions has risen from 43,364 in 2007 to 63,048 in 2015, an increase of 45%. This rise has settled at about 4.0% per annum and is in line with France (4.7%) and Australia (3.4%). However, in most other comparable countries, the rate of compulsory admissions has been steady or has declined slightly. This recent rapid rise in the UK has been associated with a substantial reduction in available beds [15]. Currently, one third of all admissions are compulsory, and a further third are converted to compulsory while the patients are in hospital. In many inner-city areas, virtually all inpatients are compulsory. It has been suggested that the current rapid rise reflects not only the risk-averse nature of UK society but also that it may be the only way to secure a bed.

The second area of concern has long been expressed and relates to the very high rates of admission (including compulsory admission) for black patients (Afro-Caribbean men in particular). This was initially attributed to stigmatizing discrimination and over diagnosis [16]. Despite careful epidemiological work to contradict this and demonstrate a genuinely high rate of psychosis in these groups [17,18], the failure of services to engage with this vulnerable group generates constant criticism.

## RESOURCES

The UK lies in the mid-range in terms of the numbers of both psychiatrists and psychiatric beds in Europe [19]. The UK has 19 psychiatrists per 100,000 population, compared with 17 for Ireland, 18 for Italy, 23 for France and Norway, and 22 for Germany and Sweden. There are 46 beds per 100,000 in the UK, compared to a European mean of 21 per 100,000 (ranging from 10 in Italy, up to 128 in Germany and 139 in the Netherlands). Average inpatient stays are about 35 days, but this mean hides a skewed curve, with many crisis admissions of two to three days and a small number of patients with very long stays. Long-Stay rehabilitation beds have been in sharp decline for the last couple of decades, but there has been a noticeable rise in secure provision (often in the private sector) for NHS forensic patients. The only three high secure forensic hospitals have been reduced by over 75% during the last two decades, while medium secure forensic units have now become a routine component of local service provision.

Figures for day hospital places (provided by the NHS) and day centre places (provided by local authorities) are difficult to obtain with any accuracy. The strong clinical impression, however, is that these are also being closed, even more so during the last decade of austerity.

## STRENGTHS AND WEAKNESSES

UK community mental health services have been the backbone of psychiatric care since WWII. They have several strengths. They have benefited from a pragmatic approach, avoiding ideological schisms. A relatively simple funding formula avoids perverse incentives and distortions of clinical care. Central monitoring and target setting have resulted in a healthy respect for, and focus on, evidence-based practice. Services have benefitted from a well-established primary care system which filters their referrals and which has taken on most of the milder cases of anxiety and depression. Another strength has been the early development of sectorized multidisciplinary teams, with an emphasis on outreach and highly trained and confident non-medical staff.

Among their weaknesses has been an absence of strong clinical leadership, with an increasingly managerial and risk-averse culture. While there have been significant improvements in the quality and consistency of care, this has been accompanied by an enormous growth in bureaucracy and a fragmentation and overcomplication of services. Simple lines of responsibility between patient, family doctor and psychiatric team have been obscured or abandoned, and continuity of care has diminished. UK community mental health services have also struggled to establish a confident and convincing public image and consequently endure problems of low morale and recruitment.

There are, however, encouraging signs that mental health issues have recently moved up the political agenda. The increasing public willingness to be open about mental health problems has focused the government's attention on patchy service provision and the gap between rhetoric and reality as regards funding. Substantially increased funding has been promised, and a review of the mental health act is underway. It would be foolish, however, to make predictions about anything in the UK currently.

## Conflict of interest

The author declares no conflict of interest.

## Funding

The author declares that there was no funding for this work.

## References

[journal-article-ref-2f4c51b3ff92279d63f47e1550fe12fe] Strathdee  G (1994). The GP, the community and shared psychiatric care. Practitioner.

[journal-article-ref-79ea08e2ac17d0a7653d040325b3d5f7] Jones  D. (1982). The Borders Mental Health Service. British Journal of Clinical & Social Psychiatry.

[journal-article-ref-637d6f752dfff658c13edab3d8ef1a1d] White Edward (1999). The 4th quinquennial national community mental health nursing census of England and Wales. Australian and New Zealand Journal of Mental Health Nursing.

[journal-article-ref-8793646a173db8f47a3c55f8ef5c4c6d] Burns  T (2000). Maxwell Jones Lecture: The legacy of therapeutic community practice in modern community mental health services. Ther Communities.

[journal-article-ref-705bbb78f24fc1ea481a4cc6965bc2d4] Stein Leonard I. (1980). Alternative to Mental Hospital Treatment. Archives of General Psychiatry.

[webpage-ref-bcbf54b2f7f87080a7dbea47c796ca2b] Department of Health and Social Care (1999). National Framework for Mental Health: Modern Standards and Service Models. https://assets.publishing.service.gov.uk/government/uploads/system/uploads/attachment_data/file/198051/National_Service_Framework_for_Mental_Health.pdf.

[journal-article-ref-ae9a0276e11c7f03f98609fc6d06a3ff] Burns Tom, Creed Francis, Fahy Tom, Thompson Simon, Tyrer Peter (1999). Intensive versus standard case management for severe psychotic illness: a randomised trial. The Lancet.

[journal-article-ref-faa1b8d4048c972f8199c56ceb11bf61] Burns Tom, Catty Jocelyn, Dash Michael, Roberts Chris, Lockwood Austin, Marshall Max (2007). Use of intensive case management to reduce time in hospital in people with severe mental illness: systematic review and meta-regression. BMJ.

[book-ref-517039f060e9dd7b4db243b244a40f7b] National Institute for Mental Health (E) (2003). Personality Disorder: No longer a diagnosis of exclusion. Policy implementation guidance for the development of servcies for people with personality disorder.

[journal-article-ref-600061d82eda387b9ed5ee78254fe51a] Burns Tom, Baggaley Martin (2017). Splitting in-patient and out-patient responsibility does not improve patient care. British Journal of Psychiatry.

[journal-article-ref-1c49a7a6095f6d526a8b74d8a31b5af2] Clark David M. (2011). Implementing NICE guidelines for the psychological treatment of depression and anxiety disorders: The IAPT experience. International Review of Psychiatry.

[journal-article-ref-01ff80b80e747d024acc0e683c59a48c] Kisely Steve, Hall Katharine (2014). An Updated Meta-Analysis of Randomized Controlled Evidence for the Effectiveness of Community Treatment Orders. The Canadian Journal of Psychiatry.

[journal-article-ref-8a819c152bc0447ebf059ac99bc334e1] Burns Tom, Rugkåsa Jorun, Molodynski Andrew, Dawson John, Yeeles Ksenija, Vazquez-Montes Maria, Voysey Merryn, Sinclair Julia, Priebe Stefan (2013). Community treatment orders for patients with psychosis (OCTET): a randomised controlled trial. The Lancet.

[journal-article-ref-1bc66c57830f5f37d81f6662dd3a0d83] Rains Luke Sheridan, Johnson Sonia I (2019). Psychiatric bed numbers in Australia – Author's reply. The Lancet Psychiatry.

[journal-article-ref-23d9857da8d9c2898cb56275b48c37dc] Keown P., Weich S., Bhui K. S., Scott J. (2011). Association between provision of mental illness beds and rate of involuntary admissions in the NHS in England 1988-2008: ecological study. BMJ.

[book-ref-209df30bdeeb130104f896cb1a943474] Littlewood  R, Lipsedge  M (1997). Aliens and Alienists: Ethnic minorities and psychiatry.

[journal-article-ref-460cb9284125255ef198ed8777e953e2] Morgan Craig, Mallett Rosemarie, Hutchinson Gerard, Bagalkote Hemant, Morgan Kevin, Fearon Paul, Dazzan Paola, Boydell Jane, McKenzie Kwame, Harrison Glynn, Murray Robin, Jones Peter, Craig Tom, Leff Julian (2005). Pathways to care and ethnicity. 1: Sample characteristics and compulsory admission. British Journal of Psychiatry.

[journal-article-ref-fa50b282ed53a1c6379fa5fe5bc94674] Singh Swaran P., Paul Moli, Parsons Helen, Burns Tom, Tyrer Peter, Fazel Seena, Deb Shoumitro, Islam Zoebia, Rugkåsa Jorun, Gajwani Ruchika, Thana Lavanya, Crawford Michael J. (2017). A prospective, quantitative study of mental health act assessments in England following the 2007 amendments to the 1983 act: did the changes fulfill their promise?. BMC Psychiatry.

[book-ref-cbaf8aead23203072a524f7cd8b90e67] World Health Organization (2018). 2017 Mental Health ATLAS.

